# ERAP1 enzyme-mediated trimming and structural analyses of MHC I–bound precursor peptides yield novel insights into antigen processing and presentation

**DOI:** 10.1074/jbc.RA119.010102

**Published:** 2019-10-10

**Authors:** Lenong Li, Mansoor Batliwala, Marlene Bouvier

**Affiliations:** Department of Microbiology and Immunology, University of Illinois, Chicago, Illinois 60612

**Keywords:** immunology, antigen presentation, antigen processing, structural biology, endoplasmic reticulum (ER), CD8+ T cells, endoplasmic reticulum aminopeptidase (ERAP), immunopeptidome, major histocompatibility complex I (MHC I), adaptive immunity, HLA-B*0801

## Abstract

Endoplasmic reticulum aminopeptidase 1 (ERAP1) and ERAP2 critically shape the major histocompatibility complex I (MHC I) immunopeptidome. The ERAPs remove N-terminal residues from antigenic precursor peptides and generate optimal-length peptides (*i.e.* 8–10-mers) to fit into the MHC class I groove. It is therefore intriguing that MHC class I molecules can present N-terminally extended peptides on the cell surface that can elicit CD8+ T-cell responses. This observation likely reflects gaps in our understanding of how antigens are processed by the ERAP enzymes. To better understand ERAPs' function in antigen processing, here we generated a nested set of N-terminally extended 10–20-mer peptides (RA)*_n_*AAKKKYCL covalently bound to the human leukocyte antigen (HLA)-B*0801. We used X-ray crystallography, thermostability assessments, and an ERAP1-trimming assay to characterize these complexes. The X-ray structures determined at 1.40–1.65 Å resolutions revealed that the residue extensions (RA)*_n_* unexpectedly protrude out of the A pocket of HLA-B*0801, whereas the AAKKKYCL core of all peptides adopts similar, bound conformations. HLA-B*0801 residue 62 was critical to open the A pocket. We also show that HLA-B*0801 and antigenic precursor peptides form stable complexes. Finally, ERAP1-mediated trimming of the MHC I–bound peptides required a minimal length of 14 amino acids. We propose a mechanistic model explaining how ERAP1-mediated trimming of MHC I–bound peptides in cells can generate peptides of canonical as well as noncanonical lengths that still serve as stable MHC I ligands. Our results provide a framework to better understand how the ERAP enzymes influence the MHC I immunopeptidome.

## Introduction

The immune recognition of surface MHC^2^ I/peptide complexes initiates activation of CD8+ T cells as a critical step in the elimination of pathogens. MHC I molecules generally bind peptides of 8–10 amino acid residues; this represents an optimal length for peptides to span the binding cleft in an elongated conformation and form stabilizing H-bond interactions with MHC residues within the A and F pockets (1, 2). Interestingly, there is increasing evidence from MS analysis that peptides with more than 11 amino acids can be eluted from human and mouse MHC I molecules (3–11). Moreover, long peptides can be presented on the surface of cells by MHC I molecules (4, 5, 7, 8) and can stimulate CD8+ T-cell responses (4, 5, 7, 8, 11–14). These can be long overlapping peptides that share much of their core sequences, nested sets of peptides that share the same core sequence and carry N- or C-terminal residue extensions, or single long peptides. These observations raise two important questions. 1) How do MHC I molecules present long peptides? 2) Have surface N-terminally extended peptides escaped trimming by the endoplasmic reticulum (ER) aminopeptidases (ERAPs)?

X-ray crystallography has revealed that peptides of 10 and 11 amino acids can fit into the MHC I groove by adopting conformations in which the middle residues zig-zag or bulge out of the groove, while still maintaining the peptide N and C termini bound within the A and F pockets, respectively (15). Peptides as long as 16-mers have been reported to adopt such highly bulged conformations (11–14, 16, 17). Structures of MHC I molecules have also shown that peptides of optimal lengths, such as 9- and 10-mers, can adopt unusual bound conformations in which a single “extra” terminal amino acid is positioned outside of the MHC I groove at the N- or C-terminal end (5, 18–23). Finally, several recent structures of MHC I molecules presenting long C-terminally extended peptides showed that the C-terminal residues exit out at the F pocket (9, 22, 24). Overall, crystallographic studies have informed us that peptides of various lengths can serve as MHC I ligands, often by adopting unconventional binding modes.

Antigenic peptides that are destined to become ligands of human MHC I molecules are generated first as long precursors by the cytosolic proteasome. These precursors are transported into the ER by the transporter associated with antigen processing (TAP), where they are then N-terminally trimmed by ERAP1 and ERAP2 (25, 26) as well as undergoing a proofreading process to assess their ability to stabilize energetically MHC I molecules (27). We and others showed that the ER-resident protein tapasin (TPN) serves as a critical checkpoint in the generation of the MHC I immunopeptidome (28–31). Importantly, studies showed that loss of ERAP1 function in humans or mice (ERAAP) leads to alterations in the MHC I immunopeptidome (32, 33). These results established ERAP1 as yet another important protein that shapes the MHC I peptide repertoire, likely working in synergy with tapasin (34). Interestingly, evidence has been provided that ERAP1 and ERAP2 can form a heterodimer (ERAP1/ERAP2) with distinct functional properties relative to the individual aminopeptidases (33, 35). Notably, in previous studies, we showed that MHC I–bound N-terminally extended peptides are trimmed by the ERAP1/ERAP2 heterodimer to optimal lengths of 8- and 9-mers (36). These results extended other studies that support the view that both free and MHC I–bound precursors are substrates of the ERAP enzymes (37–40). Therefore, it is intriguing that long N-terminally extended peptides can be presented as surface ligands by MHC I molecules, even in cells with active ERAP function. It suggests that such peptides have seemingly escaped trimming by the ERAPs in the ER and have emerged still in their precursor forms.

In this study, we used the cell-surface presentation of N-terminally extended peptides by MHC I molecules as a unique opportunity to elucidate how these peptides bind into the groove and better understand the role of the ERAPs in generating the MHC I immunopeptidome. We determined the crystal structures and thermostability of a set of N-terminally extended peptides bound to HLA-B*0801, and monitored the trimming of these peptides by ERAP1 both in their free and MHC I–bound forms. Together, our results provide novel mechanistic insights into antigen processing that explain how both canonical and noncanonical length peptides can be generated by the ERAPs and stably presented by MHC I to CD8+ T cells.

## Results

### N-terminally extended peptides

We designed a nested set of N-terminally extended peptides based on the 8-mer HIV-1 Gag immunodominant epitope GGKKKYKL (41). To eliminate possible issues with peptides dissociating from the HLA-B*0801 groove during trimming by ERAP1, we introduced a Lys-to-Cys mutation at P7 in AAKKKYKL and also introduced the complementary Glu^76^ → Cys mutation in HLA-B*0801, as we described previously (36). Based on AAKKKYCL, we produced the nested set of N-terminally extended 10-, 12-, 14-, and 20-mer peptides (RA)*_n_*AAKKKYCL that can be covalently bound to HLA-B*0801E76C. It is noteworthy that such long peptides would still bind if they were not covalently bound to the MHC I molecule (36). Finally, for the purpose of our crystallographic studies, the first N-terminal Ala residue extension was *N*-methylated (*N*-Me), generating (R(*N*-Me)A)(RA)*_n_*_− 1_AAKKKYCL. All complexes were refolded *in vitro* as described under “Experimental procedures.”

### (R(N-Me)A)AAKKKYCL protrudes out of the A pocket

**Figure 1.**
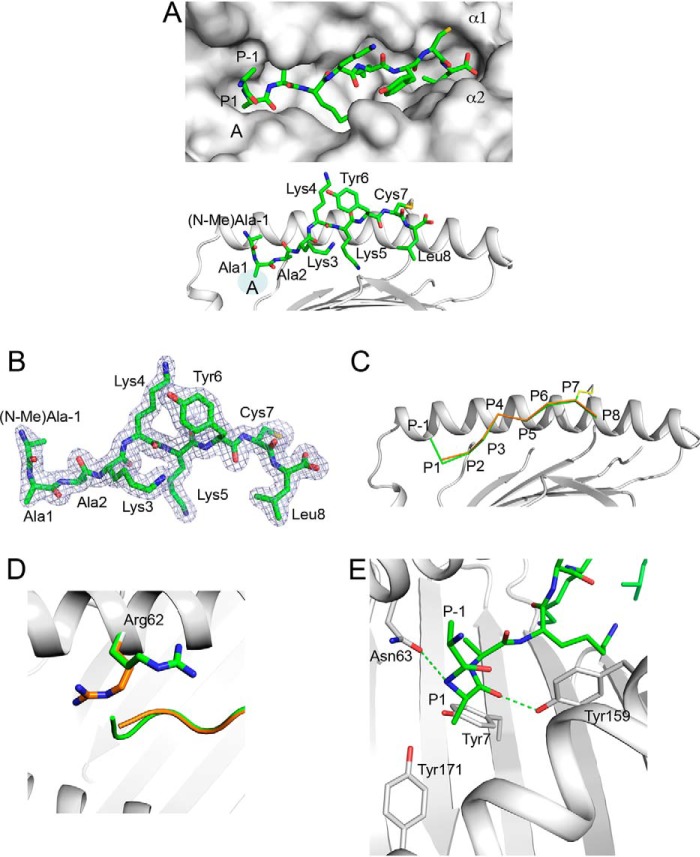
Presentation of 10-mer (R(*N*-Me)A)AAKKKYCL by HLA-B*0801E76C. *A*, *top*, molecular surface of HLA-B*0801E76C groove (*light gray*) showing the bound (R(*N*-Me)A)AAKKKYCL (*green*) with P−1 (*N*-Me)Ala and P−2 Arg (see *B*) extending out of the A pocket. Peptide residue positions (*P*), A pocket, and α-helices are indicated. *Bottom*, bound conformation of (R(*N*-Me)A)AAKKKYCL (*green*) shown against the α1-helix of HLA-B*0801E76C (*light gray*); the AAKKKYCL core binds into the groove, whereas P−1 (*N*-Me)Ala and P−2 Arg (see *B*) protrude out. The disulfide bond (*yellow*) between P7 Cys and Cys^76^ is shown. Peptide residues are *labeled. B*, 2m*F_o_*-D*F_c_* electron density, contoured at 1σ, is shown as *blue mesh* around (R(*N*-Me)A)AAKKKYCL. The density is visible for all residues except for the *N*-methyl group of P−1 (*N*-Me)Ala and P−2 Arg. *C*, superimposition of the canonical structure of HLA-B*0801–bound GGKKKYKL (*orange*) (PDB entry 1AGD) with the structure of HLA-B*0801E76C–bound (R(*N*-Me)A)AAKKKYCL (*green*), shown against the α1-helix of HLA-B*0801E76C (*light gray*). *D*, same as in *C*, showing that in the canonical structure of HLA-B*0801–bound GGKKKYKL (*orange*), Arg^62^ occupies a position that blocks the A pocket. In contrast, in the structure of HLA-B*0801E76C-bound (R(*N*-Me)A)AAKKKYCL (*green*), Arg^62^ has moved up and out, which opens the A pocket and allows the extensions to protrude out. *E*, details of interactions within the A pocket showing rotation of P1 Ala and its effect on P−1 (*N*-Me)Ala position. The H-bonds between the main-chain nitrogen of P1 Ala and Asn^63^ and between the main-chain carbonyl oxygen of P1 Ala and Tyr^159^ are shown as *green dashed lines*.

**Figure 2.**
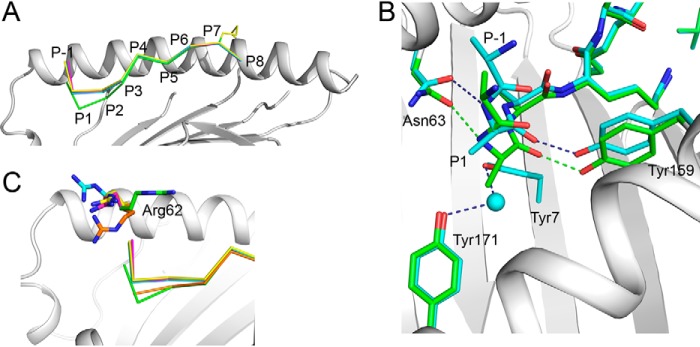
Binding of a nested set of N-terminally extended peptides (R(*N*-Me)A)(RA)*_n_*_-1_AAKKKYCL into HLA-B*0801E76C. *A*, superimposition of the structures of 10-mer (*green*), 12-mer (*cyan*), 14-mer (*magenta*), and 20-mer (*yellow*) (R(*N*-Me)A)(RA)*_n_*_-1_AAKKKYCL, shown against the α1-helix of HLA-B*0801E76C/10-mer complex (*light gray*). The backbone conformations of the peptides are very similar except between P1 and P3. *B*, superimposition of the structures of the 10-mer (*green*) and 12-mer (*cyan*) (R(*N*-Me)A)(RA)*_n_*_-1_AAKKKYCL, shown within the A pocket of HLA-B*0801E76C/10-mer complex (*light gray*). A water molecule (*cyan*) occupies the A pocket in the 12-mer structure. Hydrogen bonds are shown as *green* and *dark blue dashed lines* for the 10- and 12-mer, respectively. *C*, same as in *A*, showing that in the canonical structure of HLA-B*0801-bound GGKKKYKL (*orange*), the configuration of Arg^62^ “closes” the A pocket. Arg^62^ changes its position significantly upon binding the 10-mer (*green*) (see also Fig. 1*D*), 12-mer (*cyan*), 14-mer (*magenta*), and 20-mer (*yellow*).

To understand how a N-terminally extended 10-mer peptide is presented by HLA-B*0801, we determined the X-ray crystal structure of (R(*N*-Me)A)AAKKKYCL covalently bound to HLA-B*0801E76C at 1.65 Å (Table 1). The structure surprisingly shows that the peptide adopts an elongated conformation in which the AAKKKYCL core is bound into the groove, and the residue extensions P−1 (*N*-Me)Ala (one position N-terminal of P1) and P−2 Arg protrude out at the A pocket (Fig. 1*A*). The electron density was well-defined over the entire length of the peptide, including the extension P−1 (*N*-Me)Ala, although no electron density at an acceptable 1σ threshold could be discerned for the *N*-methyl group of P−1 (*N*-Me)Ala and P−2 Arg (Fig. 1*B*). The P−2 Arg residue was therefore omitted in our model. Comparisons with a previously reported canonical structure of GGKKKYKL bound to HLA-B*0801 (42) showed that the peptide backbones adopt nearly identical conformations, except for a small 0.71-Å shift in P1 Cα-atom positions (Fig. 1*C*). Remarkably, only minor structural changes in the binding groove were detected between the two structures (r.m.s. deviation of 0.46 Å, over 1–180 Cα positions), except for residue Arg^62^. In the canonical structure of GGKKKYKL, the side chain of Arg^62^ acts as a lid atop of the peptide N terminus (Fig. 1*D*), whereas in our structure, this residue swings out and up, which “opens” the A pocket. In this “open” configuration, P−1 (*N*-Me)Ala and P−2 Arg can extend straight out of the groove. Thus, Arg^62^ appears to control the configuration of the A pocket between an “open” and “closed” form (see also the legend to Fig. 2*C*).

**Table 1:** Data collection and refinement statistics for HLA-B*0801E76C complexes

	10-mer	12-mer	14-mer	20-mer	14-mer mutant
**Data collection**					
Wavelength	0.97872	0.97872	0.97872	0.97872	0.97872
Space group	P212121	P212121	P212121	P212121	P212121
Cell dimensions					
*a*, *b*, *c* (Å)	50.6, 80.9, 108.7	50.5, 81.4, 110.6	50.6, 81.3, 110.7	50.8, 81.8, 111.9	50.8, 81.9, 111.1
α, β, γ (degrees)	90		90	90	90
Resolution (Å)	64.91–1.65	19.66–1.59	20.0–1.59	37.6–1.40	19.21–1.48
	(1.69–1.65)*^a^*	(1.65–1.59)	(1.65–1.59)	(1.45–1.40)	(1.53–1.48)
*R*_merge_	0.072	0.053	0.052	0.06	0.07
*I*/σ*I*	13.6 (1.7)	7.2 (1.5)	22.1 (3.7)	19.1 (2.5)	11.67 (1.58)
Completeness (%)	98.9 (93.4)	99.5 (95.2)	99.3 (96.0)	99.9 (99.6)	98.8 (93.9)
Redundancy	5.7 (4.6)	3.6 (2.9)	8.1 (6.5)	9.2 (8.8)	5.6 (4.5)
**Refinement**					
No. of reflections	51,165	61,192	61,436	93,207	76,357
*R*_work_/*R*_free_	20.4/21.4 (43.5/44.7)	19.5/22.7 (29.9/34.5)	19.2/21.3 (22.5/26.5)	19.6/21.0 (23.9/25.6)	21.4/24.5 (29.5/32.3)
No. of atoms					
Protein	3147	3139	3147	3139	3142
Water	243	261	236	311	274
*B*-factors					
Protein	33.9	21.4	20.2	19.6	25.6
Water	38.4	27.1	24.9	26.9	32.7
r.m.s. deviations					
Bond lengths (Å)	0.032	0.009	0.006	0.005	0.011
Bond angles (degrees)	2.21	1.3	1.07	1.02	1.35
Ramachandran plot					
Most favored regions (%)	98.1	98.7	98.4	99.0	98.7
Allowed regions (%)	1.9	1.3	1.6	1.0	1.3
Disallowed regions (%)	0	0	0	0	0

*^a^* Values in parentheses are for the highest-resolution shell.

A close-up view of the A pocket shows that the side-chain methyl group of P1 Ala is rotated and occupies the position of a canonical N-terminal group of bound peptide structures (Fig. 1*E*). Consequently, the main-chain nitrogen of P1 Ala adopts a position corresponding to a P1 side chain normally seen in MHC I structures (Fig. 1*E*). Similar P1 residue rotations within the A pocket have been reported previously (5, 18, 43). In this unusual configuration, the two peptide residue extensions follow an upward trajectory out of the groove. In our structure, because of the lack of a peptide N-terminal group, there are noticeable changes to the conventional network of H-bonds normally seen within the A pocket (1) (Fig. 1*E*): H-bonds to conserved Tyr^7^ and Tyr^171^ residues are absent, and a new H-bond is formed between the main-chain nitrogen of P1 Ala and Asn^63^. The main-chain carbonyl oxygen of P1 Ala also forms a key H-bond with Tyr^159^ (Fig. 1*E*).

### Structures of a nested set of N-terminally extended peptides

To extend the above analysis, we determined the structures of the 12-, 14-, and 20-mer peptides (R(*N*-Me)A)(RA)*_n_*_− 1_AAKKKYCL covalently bound to HLA-B*0801E76C (Table 1). The structures show that the 8-amino acid AAKKKYCL core of all peptides, even the very long 20-mer, zigzags in the groove in an almost identical manner to the 10-mer (Fig. 2*A*). There was, however, a noticeable upward shift of the backbone from P1 to P3 for the 12-, 14-, and 20-mers relative to the 10-mer (Fig. 2*A*): Cα-atom shifts of ∼2.0 Å at P1 and ∼0.44 Å at P3. The electron density for all peptides was well-defined over the AAKKKYCL core and P−1 Ala (Fig. S1, *left panels*). It is noteworthy that partial electron density for P−2 Arg was visible at 0.5σ for the 12-, 14-, and 20-mers (Fig. S1, *right panels*), which was not the case, however, for the 10-mer. Nonetheless, we have omitted all residues starting at P−2 Arg from our final models. The structures also showed that all P1 Ala residues had undergone a rotation within the A pocket, as exemplified in Fig. 2*B* with the 12-mer. Furthermore, in all structures, because P1 Ala residues are less deeply anchored within the A pocket relative to the 10-mer (Fig. 2*B*), this created a large cavity that was filled by a water molecule. This water molecule makes the conventional H-bonds to Tyr^7^ and Tyr^171^ (Fig. 2*B*). The other H-bonds are the same as those seen in the 10-mer structure (compare with Fig. 1*E*). Notably, the structures also showed that the most significant structural change in the groove, relative to the canonical structure of GGKKKYKL, involves Arg^62^. In the 12-, 14-, and 20-mer structures, Arg^62^ swings out and up from its canonical position, which opens the A pocket (Fig. 2*C*). Although the position of Arg^62^ in the longer peptides is different from that seen in the 10-mer, and small differences are seen between the 12-, 14-, and 20-mers, all orientations generated an open-ended groove. Thus, these structures further support the view that Arg^62^ plays a critical role in modulating the “open” and “closed” forms of the A pocket.

### Role of P−5 (N-Me)Ala and P5 middle anchor residue in the MHC I–bound conformation of (R(N-Me)A)(RA)_2_AAKKKYCL

**Figure 3.**
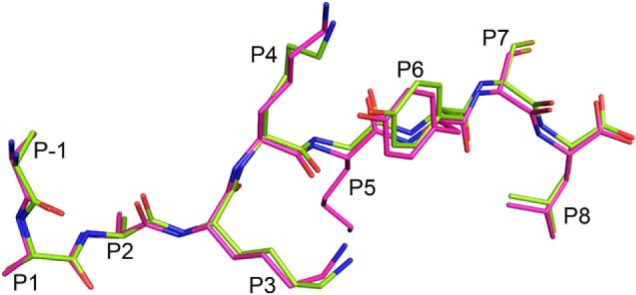
**Examining the effect of P−5 (*N*-Me)Ala and P5 middle anchor residues.** Superimposition of the HLA-B*0801E76C–bound 14-mers (RA)_3_AAKKGYCL (*lime*) and (R(*N*-Me)A)(RA)_2_AAKKKYCL (*magenta*). The two peptides bind in a nearly identical manner and share very similar structural features. The peptide residue positions (*P*) are indicated.

We wanted to verify that the *N*-methylation of the N-terminal Ala residue and the presence of the middle anchor residue at P5 were not controlling how the extensions (R(*N*-Me)A)(RA)*_n_*_-1_ protrude out of the A pocket. For this, we generated a 14-mer mutant in which P−5 is occupied by an Ala residue and the P5 anchor residue is mutated to Gly (*i.e.* (RA)_3_AAKKGYCL). The mutant peptide was refolded with HLA-B*0801E76C, and the structure of the complex was determined to 1.48 Å (Fig. 3 and Table 1). The structure shows that the AAKKGYCL core adopts an extended bound conformation with the (RA)_3_ extensions protruding out of the A pocket (Fig. 3). Comparisons with the structure of the 14-mer (R(*N*-Me)A)(RA)_2_AAKKKYCL (Fig. 3) show that both peptides overlap fairly well and have very similar structural features, including the P1 Ala rotation. Thus, we conclude that the bound conformations of the N-terminally extended peptides, with residues extending out the groove, are not governed by the N-terminal (*N*-Me)Ala or middle anchor residues.

### Stability of N-terminally extended peptides bound to HLA-B*0801E76C

**Figure 4.**
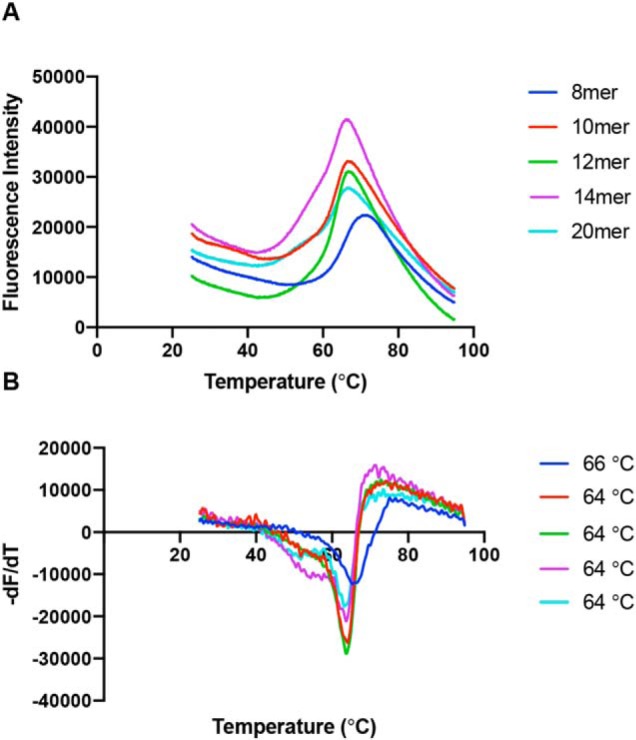
**Thermal denaturation assay.** *A*, averaged thermal denaturation curves for individual peptides, as indicated, presented by HLA-B*0801E76C. *B*, graphs of the first derivative of the curves shown in *A*. The *T_m_* values, determined from the minima, are indicated for each peptide. The *T_m_* value of each complex was unchanged with different refolding batches.

To assess the stability of HLA-B*0801E76C complexes loaded with long peptides, we carried out a thermal denaturation assay based on differential scanning fluorometry. For this, we determined the stability of the 8-mer AAKKKYCL covalently bound to HLA-B*0801E76C and compared it with that of the 10-, 12-, 14-, and 20-mer peptides (RA)*_n_*AAKKKYCL (Fig. 4*A*). The melting temperature (*T_m_*) of the 8-mer was 66 °C, and only slightly lower *T_m_* values of 64 °C were determined for the 10-, 12-, 14-, and 20-mers (Fig. 4*B*). These results suggest that the N-terminally extended peptides have surprisingly similar stabilities compared with the 8-mer. This is consistent with the structures showing that the AAKKKYCL core adopts similar bound conformations within the groove (see Fig. 2*A*). Finally, we found that both AAKKKYKL and AAKKKYCL had the same *T_m_* value of 66 °C, indicating that the covalently bound nature of our peptides does not alter MHC I stability.

### Processing of free and MHC I–bound peptides by ERAP1

**Figure 5.**
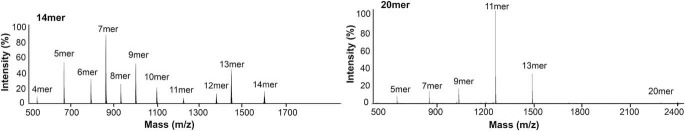
**ERAP1-mediated trimming of free 14-mer (RA)_3_AAKKKYCL and 20-mer (RA)_6_AAKKKYCL.** Free peptides (∼12 μg) were incubated with 0.4 μg of ERAP1 at 37 °C. An aliquot was taken after 20 min (14-mer) and 30 min (20-mer) and analyzed by MS. The starting peptides and their fragments are indicated.

We incubated the free and MHC I–bound 14- and 20-mer (RA)*_n_*AAKKKYCL with ERAP1 to assess the susceptibility of these peptides to processing. MS analysis of the free 14-mer (RA)_3_AAKKKYCL shows that after 20 min of trimming by ERAP1, the mixture contained a series of various fragments as short as 4-mer (Fig. 5). In contrast, and under identical conditions, ERAP1 trimmed the free 20-mer (RA)_6_AAKKKYCL to predominantly the 11-mer (Fig. 5). It is worth noting that ERAP1 was unable to trim a different free 20-mer (RA)_6_AAKKKYKL.^3^ We attribute the inconsistencies in trimming free 20-mers to that random secondary structures that such long peptides likely adopt in solution.

**Figure 6.**
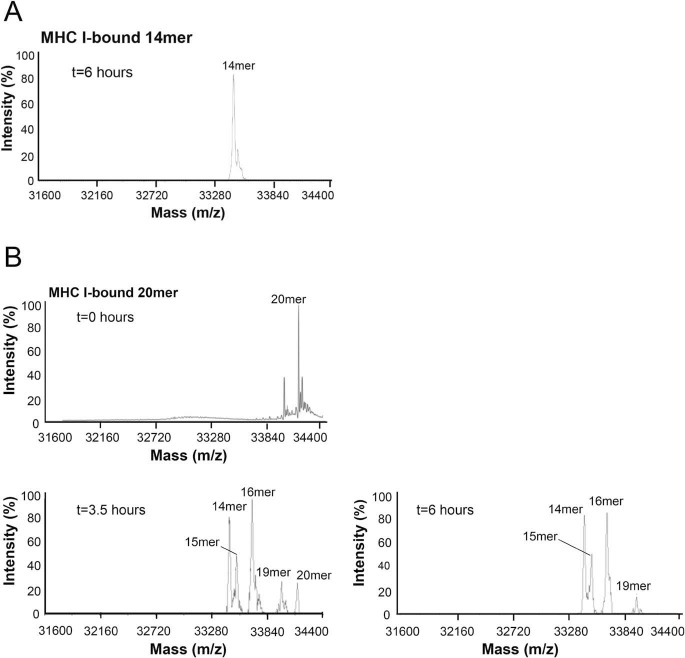
ERAP1-mediated trimming of HLA-B*0801E76C-bound peptides. MHC I–bound 14-mer (RA)_3_AAKKKYCL (*A*) and 20-mer (RA)_6_AAKKKYCL (∼12 μg of peptide) (*B*) were incubated with ∼34 μg of ERAP1 at 37 °C. Aliquots were taken from the mixtures at the indicated times and analyzed by MS. Additional ERAP1 was added to the mixtures after the aliquots of 3.5 and 6 h. The starting MHC I–bound peptides and fragments are indicated.

Next, we examined the ability of ERAP1 to trim the MHC I–covalently bound 14-mer (RA)_3_AAKKKYCL. Results from MS analysis show that ERAP1 was unable to trim the bound 14-mer over a period of 6 h (Fig. 6*A*), even with additional ERAP1 and trimming continued to 10 h.^3^ These results are in marked contrast to those obtained for the free 14-mer, which was readily trimmed by ERAP1 to small fragments (see Fig. 5). We then monitored the ability of ERAP1 to trim the MHC I–bound 20-mer (RA)_6_AAKKKYCL (Fig. 6*B*, *t* = 0). MS analysis of the mixture after 3.5 h showed that the major products were the covalently bound 14-, 15-, and 16-mer. After 6 h and with additional ERAP1, the remaining 20-mer was absent in the mixture, while the relative abundance of the other fragments was largely unchanged. The mixture composition remained the same even after 10 h.^3^ Overall, these results are consistent and show that trimming of the MHC I–covalently bound 20-mer generated the 14-mer as the shortest covalently linked peptide (*i.e.* the same molecular species that was resistant to trimming when used as a starting MHC I–bound peptide).

### Model of MHC I–bound long peptides

**Figure 7.**
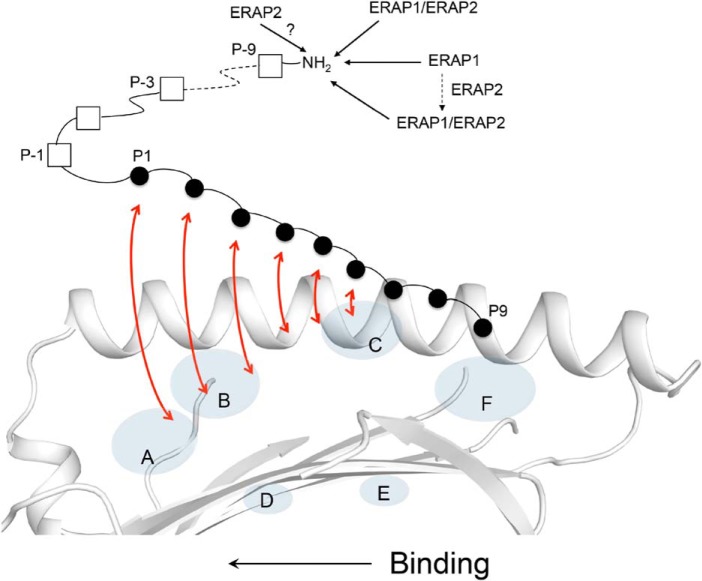
**Trimming of MHC I–bound 18-mer precursor peptide by the ERAP enzymes.** Shown is a model of antigen processing in which an N-terminally extended candidate peptide is bound into the MHC I groove by only a few C-terminal residues. As the peptide undergoes a dynamic binding and “sampling” into the groove (indicated by *red arrows*), from its C to N terminus, the N-terminal residue extensions are concurrently trimmed by the ERAPs. Inside the cells, the ERAP1 and ERAP2 enzymes likely exist in more than one molecular form, with each form shaping differently the MHC I immunopeptidome (see “Discussion”). *ERAP1/ERAP2* indicates the heterodimer.

The results obtained here help refine our model of how N-terminally extended peptides can bind into the MHC I groove (Fig. 7) (36). We propose that precursor peptides are anchored into the groove by a few C terminus residues with their remaining N-terminal residues extending into the solvent space. This binding mode is supported by data showing the importance of anchoring the C-terminal peptide residue within the F pocket (44, 45). Interestingly, the structures of TAPBPR (TAP-binding protein–related), a TPN-like protein, in complex with MHC I showed that a conformationally flexible “scoop” loop of TAPBPR is positioned into the F pocket region of the MHC I groove; this feature provides further evidence that the C terminus of the peptide is critical during the initial stage of binding (and editing) (46, 47). Finally, that the N terminus of MHC I–bound peptides can dissociate partially from the groove is supported by molecular dynamics simulations of MHC I/peptide complexes (40). Thus, taken together, we suggest that as precursor peptides bind to MHC I, from their C- to N-terminal ends, the N-terminal extensions can be concomitantly trimmed by the ERAP enzymes (see “Discussion”).

## Discussion

It is increasingly evident that the MHC I immunopeptidome is more diverse in peptide lengths than originally thought, and that peptides of up to 20 amino acids or more can be presented by MHC I and elicit immune responses. Our objective was to demonstrate how a nested set of unusually long N-terminally extended peptides are presented by MHC I as well as to take advantage of the unique opportunity offered by such MHC I ligands to fill gaps in our understanding of how the ERAP enzymes shape the MHC I immunopeptidome.

Our structures revealed that the N-terminally extended peptides bind into the MHC I groove by adopting noncanonical conformations with residue extensions protruding out of the A pocket. Our structures also showed that the 8-amino acid core sequence of all peptides overlap fairly well into the groove. Residue Arg^62^ was critical to open the A pocket in response to binding long peptides; whether or not other factors govern opening of the A pocket remains to be determined. Our analysis suggests that there is no apparent limit in peptide lengths that would prevent a given N-terminally extended peptide to be presented by HLA-B*0801. This is consistent with our thermostability data showing that N-terminally extended peptides form remarkably stable complexes. Overall, our study provides a structural and biochemical framework to understand how N-terminally extended peptides can be stably presented by MHC I on the surface of cells.

We compared our structures with the recent structures of the HIV-1 Gag immunodominant epitope TSTLQEQIGW (TW10) presented by HLA-B*5701 and HLA-B*5801 (5, 19). In these structures, the side chain of Ser occupied the canonical position of a P1 peptide N-terminal group within the A pocket, whereas its main-chain nitrogen occupied the canonical position of a P1 side chain; these structural features are comparable with our P1 Ala residues. Because of this P1 rotation, the N-terminal Thr of TW10 acted as an “extra” P−1 residue by exiting out of the A pocket, in a similar fashion to the P−1 residues of our peptides. Thus, overall, these three structures illustrate how peptides of different lengths bound to three different HLA-B molecules use the same molecular mechanism involving changes in the P1 peptide position to allow N-terminally extended peptides to exit out of the A pocket. Because both HLA-B*5701 and HLA-B*5801 have a Gly residue at 62, it was not possible to assess whether this residue was critical in allowing TW10 P−1 Thr to exit out of the groove. It is, however, noteworthy that residue Trp^62^ was shown to play a critical role in the extension of long peptides out of the HLA-F groove (48). Interestingly, an analysis of several HLA-A, -B, and -C structures presenting different 9-mer peptides showed that polymorphic residue 62 changes its configuration, depending on the size of the peptide side chain at P1. Thus, on that basis, we suggest that HLA-A and -C molecules have the potential to present long N-terminally extended peptides. Finally, recent structures of HLA-A*0201 presenting C-terminally extended peptides showed that these peptides bind with their residue extensions exiting at the F pocket (9, 24). In these structures, as seen in our study, the normally “closed” configuration of the F pocket became “open” from the movement of a single residue, namely Tyr^84^ or Lys^146^. Overall, we suggest that as more surface N- and C-terminally extended peptides are identified and characterized, it will be evident that such “unconventional” MHC I–bound conformations in which peptides exit at one end of the groove are more common than is currently realized.

We also showed that the MHC I–bound 20-mer peptide was trimmed by ERAP1 to the 14-mer as the shortest final peptide, and this result was corroborated by the inability of ERAP1 to trim the starting MHC I–bound 14-mer. Thus, there is a positive correlation between efficiency of cleavage by ERAP1 and length of peptides, where at least 5–6 amino acids have to extend out of the A pocket, based on peptide conformations seen in our structures, to be accessible by ERAP1. These experimental data support a modeling exercise based on the X-ray structure of ERAP1 that predicted that 6 peptide residues would need to protrude out of the groove to reach the zinc active site (49).

Taken together, we propose the following model for the trimming of MHC I–bound peptides by the ERAP enzymes (Fig. 7). We suggest that as a candidate precursor peptide “lands” into the MHC I groove by its C-terminal residues, the N-terminal residue extensions are solvent-exposed and thus susceptible to trimming by the ERAPs (Fig. 7). As the N-terminal extensions are being trimmed, the peptide undergoes a dynamic binding from its C to N terminus (50–52) and “sampling” (editing), possibly in the presence of MHC I accessory proteins (also see below). The editing of candidate peptides ensures that only peptides that meet a given threshold of stabilization energy become ligands of MHC I (29). We also suggest that whether the final MHC I complex will present an optimal-length peptide or a peptide that is still in its precursor form depends largely on which ERAP molecular species is engaged in processing the partially bound precursor (Fig. 7). Based on our results, ERAP1 is more likely to yield peptides of noncanonical lengths, such as nested sets of N-terminally extended peptides that share a common core of 8 or 9 residues stably anchored into the groove, as shown in our structures. This view takes into consideration the peptide length dependence exhibited by ERAP1 that prevents removal of all residue extensions. On the other hand, as we showed previously (36), the ERAP1/ERAP2 heterodimer is more likely to generate peptides of canonical lengths (see also below). In this scenario, the relative rate of peptide binding/“sampling” *versus* the rate of peptide trimming by the ERAPs is also likely to influence the final lengths of the bound peptides.

The recently determined cryo-EM structure of the peptide-loading complex (PLC) showed for the first time that TPN, calreticulin, ERp57, and MHC I are arranged in a pseudosymmetric manner around the transporter TAP (53). In this macromolecular organization, the N terminus of the MHC I groove is positioned away from the center of the PLC (*i.e.* at the outer surface of the assembly). Based on this, it seems sterically possible for the ERAP aminopeptidases to approach the PLC and access precursor peptides that are partially bound into MHC I molecules, as depicted in Fig. 7. Furthermore, from a kinetics perspective, it would be more efficient if the transported precursors that bind into PLC-engaged MHC I molecules are trimmed by the ERAPs while they are still bound in the editing module, rather than for the free precursors diffusing away from the PLC for a chance encounter with an aminopeptidase. Thus, the PLC structure is consistent with the trimming of precursor peptides that are partially bound into PLC-engaged MHC I molecules.

To date, the intermolecular relationship between ERAP1 and ERAP2 is undefined. Hence, it is unclear how ERAP1 working with ERAP2, in contrast to ERAP1 alone, can yield MHC I–bound peptides of canonical lengths. This could be due to ERAP2 altering the structure/function of ERAP1, a molecular interplay between ERAP2 and MHC I that enhances exposure of peptide residue extensions to ERAP1, or some other reasons to be determined. The functional interdependency of ERAP1 and ERAP2, and whether the enzymes are only transiently active as a heterodimer or not, is also unclear. It is also undetermined whether ERAP2 trims MHC I–bound peptides (Fig. 7). Given that the expression levels of ERAP1 and ERAP2 vary with cell subsets (26), and not all individuals even express ERAP2 (54), these are important questions to address in future studies.

In summary, our study has provided new basic knowledge to understand how the processing of MHC I–bound precursor peptides by different molecular forms of the ERAP enzymes generate final peptides of both canonical and noncanonical lengths that contribute to the natural length distribution of the MHC I immunopeptidome. We also provided a structural and biochemical basis to explain how long peptides form stable MHC I complexes and thus can be stably presented on the cell surface. As we more fully appreciate the cell-surface presentation of unusually long MHC I peptides, a next goal is to develop a better understanding of how such structures are recognized by CD8+ T cells.

## Experimental procedures

### Synthetic peptides

Peptides were synthesized by the solid-phase methodology on a Symphony synthesizer (Protein Technologies Inc.) and purified by reverse-phase HPLC on a C18 Agilent column. Stock solutions in DMSO (∼10 mg/ml) were stored at −80 °C.

### Refolding of HLA-B*0801E76C complexes

We examined the crystal structure of HLA-B*0801/GGRKKYKL (PDB code 1AGB) to identify a residue in HLA-B* 0801 heavy chain (Glu^76^) that is geometrically well-positioned to form a disulfide bond linkage with a peptide residue side chain, upon mutation with a cysteine, as we described previously (36). The HLA-B* 0801E76C heavy chain mutant was generated as described previously (36). HLA-B*0801E76C complexes were reconstituted from urea-solubilized inclusion bodies of HLA-B*0801E76C heavy chain (1 μm) and β_2_-microglobulin (2 μm) with a synthetic peptide (10 μm) in an oxidative refolding buffer (55). The crude refolding mixtures of HLA-B*0801E76C complexes were purified on a Superdex 200 gel-filtration column by FPLC. Stock solutions of purified complexes (10–30 mg/ml) in 20 mm Tris-HCl, pH 7.5, 150 mm NaCl were kept at −80 °C.

### Baculovirus expression system for ERAP1

The plasmid of full-length human ERAP1 cloned into the pFastBac vector containing a tobacco etch virus (TEV) protease cleavable C-terminal 10× histidine tag (TEV-His_10_-FLAG) was a gift from Addgene (Addgene plasmid no. 39174; RRID:Addgene_39174). The generation of recombinant baculovirus for the expression of recombinant ERAP1 was carried out using the Bac-to-Bac baculovirus expression system (Invitrogen) as recommended by the manufacturer.

### Expression and purification of ERAP1

High Five insect cells were cultured at 27 °C in serum-free Express Five medium (Gibco) supplemented with 0.75% fetal bovine serum (Gibco). Infection of High Five cells with recombinant baculovirus was carried out in 2-liter Delong flasks at a cell density of 1.8–2 × 10^6^ cells/ml, followed by gentle shaking at 27 °C for 70 h. The culture medium (3 liters) was centrifuged (6500 × *g*, 25 min, 4 °C), and the supernatants were supplemented with 10% glycerol followed by concentration to 300 ml (10-fold) using a Prep/scale-TFF cartridge (Millipore, Burlington, MA). The concentrated supernatant was dialyzed overnight in 50 mm Tris-HCl, pH 8.0, 10 mm imidazole, 10% glycerol, and 300 mm NaCl. The supernatant was then loaded onto a nickel-nitrilotriacetic acid column and washed several times with 50 mm Tris-HCl, pH 8.0, 30 mm imidazole, 300 mm NaCl. The protein was eluted with 3–5 ml of 50 mm Tris-HCl, pH 8.0, 250 mm imidazole, 10% glycerol, 300 mm NaCl. The eluate was immediately supplemented with 1 mm DTT, followed by purification on a Superdex 200 gel filtration column by FPLC. The purified protein in 20 mm Tris-HCl, pH 7.5, 10% glycerol, 150 mm NaCl, 1 mm DTT was kept at −80 °C.

### Crystallization

The initial crystallization condition for HLA-B*0801E76C/(R(*N*-Me)A)AAKKKYCL (∼10 mg/ml) was identified using the Crystal Screen I (Hampton Research, Riverside, CA) as solution #15, 0.2 m ammonium sulfate, 0.1 m sodium cacodylate trihydrate, pH 6.5, 30% (w/v) PEG 8000, via the hanging-drop vapor diffusion method at room temperature. Initial crystals were optimized using different pH values (4.5–7.0) and different PEGs (10–30%; 6000–8000). A seeding solution in solution #15 was generated from these optimized crystals. Crystals used for data collection were grown by mixing 2 μl of ∼10 mg/ml protein solution with 2 μl of 0.2 m ammonium sulfate, 18% PEG 4000, 0.1 m sodium cacodylate, pH 5.7, and 0.5 μl of seeding solution. Similar crystallization conditions were used to collect data for complexes involving the 12-, 14-, and 20-mer peptides.

### Data collection, structural determination, and refinement

X-ray diffraction data sets were collected with a MAR-225 CCD detector at the LS-CAT beamline 21-ID-F (or 21-ID-G) of the Advanced Photon Source (Argonne National Laboratory, Argonne, IL). Data were integrated and scaled with the HKL-2000 program package (56) or XDS (57). Details of data processing are indicated in Table 1. The structures of all complexes were solved by molecular replacement using Phaser (58) (the initial search model was HLA-B*0801/GGRKKYKL (PDB code 1AGB). Structure refinement of all models was carried out in Phenix (or Refmac in CCP4) (59–61) and manual building with COOT (62). Final refinement statistics are summarized in Table 1. The atomic coordinates of all structures have been deposited in the Protein Data Bank with the following accession codes: 10-mer (6P2S), 12-mer (6P23), 14-mer (6P27), 20-mer (6P2C), and 14-mer mutant (6P2F).

### Thermal denaturation assay

A thermal shift assay was performed using an ABI ViiA7 RT-PCR instrument (Life Technologies, Inc., Carlsbad, CA). Reaction mixtures (total volume of 21 μl) consisted of 7 μl of complex (final concentration of 2 μm), 7 μl of 10× SYPRO orange dye (5000×, Thermo Fisher Scientific, Waltham, MA), and 7 μl of buffer 50 mm HEPES, pH 7.2, 150 mm NaCl. Each complex was analyzed in quadruplicate. A temperature gradient from 25 to 85 °C with continuous increment of 0.06 °C/s was used to generate the denaturation curves. The averaged denaturation curves were plotted as fluorescence intensity *versus* temperature. The minimum point of the first derivative of each curve provided the melting temperature (*T_m_*).

### Peptide trimming assay

The trimming of free and MHC I–bound peptides (∼12 μg) was carried out by incubating with ERAP1 (∼0.4 or ∼34 μg, respectively) in 50 mm Tris-HCl, pH 7.6, 150 mm NaCl, 100 μm ZnCl_2_, supplemented with 1 mm DTT (omitted for trimming MHC I–bound peptides), at 37 °C (total volume was 45 μl). At various times, the assay mixtures were spun down for 3 min in a microcentrifuge (14,000 × *g*), and aliquots (10 μl) were taken from the supernatants, followed by quenching with formic acid. The aliquots were kept frozen until MS analyses. In some experiments with MHC I–bound precursors, additional ERAP1 (1–17 μg) was added to the reaction mixtures after each aliquot. Each peptide was assayed 2–4 times, using different batches of ERAP1. The samples were analyzed by electrospray ionization and MALDI MS using an Agilent AJS-ESI QTOF 6545 system at the MS Core within the UIC Research Resources Center.

## Author contributions

L. L. and M. Batliwala data curation; L. L., M. Batliwala, and M. Bouvier formal analysis; L. L. and M. Batliwala validation; L. L. and M. Batliwala methodology; L. L. and M. Batliwala writing-review and editing; M. Bouvier conceptualization; M. Bouvier supervision; M. Bouvier funding acquisition; M. Bouvier writing-original draft.

## Supplementary Material

Supporting Information

## Footnotes

3 L. Li, M. Batliwala, and M. Bouvier, unpublished observations.

## Footnotes

2 The abbreviations used are:

MHC: major histocompatibility complex
ER: endoplasmic reticulum
ERAP: endoplasmic reticulum aminopeptidase
TPN: tapasin
r.m.s.: root mean square
TAP: transporter associated with antigen processing
TAPBPR: TAP-binding protein–related
PLC: peptide-loading complex
PDB: Protein Data Bank.

## References

[1] Madden D. R. (1995) The three-dimensional structures of peptide-MHC complexes. Annu. Rev. Immunol. 13, 587–622 10.1146/annurev.iy.13.040195.003103 7612235

[2] Bouvier M., and Wiley D. C. (1994) Importance of peptide amino acid and carboxyl termini to the stability of MHC molecules. Science 265, 398–402 10.1126/science.8023162 8023162

[3] Escobar H., Crockett D. K., Reyes-Vargas E., Baena A., Rockwood A. L., Jensen P. E., and Delgado J. C. (2008) Large scale mass spectrometric profiling of peptides eluted from HLA molecules reveals N-terminal-extended peptide motifs. J. Immunol. 181, 4874–4882 10.4049/jimmunol.181.7.4874 18802091

[4] Samino Y., Lopez D., Guil S., de León P., and Del Val M. (2004) An endogenous HIV envelope-derived peptide without the terminal NH_3_^+^ group anchor is physiologically presented by major histocompatibility complex MHC I molecules. J. Biol. Chem. 279, 1151–1160 10.1074/jbc.M305343200 14583622

[5] Pymm P., Illing P. T., Ramarathinam S. H., O'Connor G. M., Hughes V. A., Hitchen C., Price D. A., Ho B. K., McVicar D. W., Brooks A. G., Purcell A. W., Rossjohn J., and Vivian J. P. (2017) MHC-I peptides get out of the groove and enable a novel mechanism of HIV-1 escape. Nat. Struct. Mol. Biol. 24, 387–394 10.1038/nsmb.3381 28218747 PMC7900914

[6] Trolle T., McMurtrey C. P., Sidney J., Bardet W., Osborn S. C., Kaever T., Sette A., Hildebrand W. H., Nielsen M., and Peters B. (2016) The length distribution of class I-restricted T-cell epitope is determined by both peptide supply and MHC allele-specific binding preference. J. Immunol. 196, 1480–1487 10.4049/jimmunol.1501721 26783342 PMC4744552

[7] Yaciuk J. C., Skaley M., Bardet W., Schafer F., Mojsilovic D., Cate S., Stewart C. J., McMurtrey C., Jackson K. W., Buchli R., Olivera A., Cedeño S., Plana M., Mothe B., Brander C., West J. T., and Hildebrand W. H. (2014) Direct interrogation of viral peptides presented by the class I HLA of HIV-infected T cells. J. Virol. 88, 12992–13004 10.1128/JVI.01914-14 25165114 PMC4249081

[8] Rucevic M., Kourjian G., Boucau J., Blatnik R., Garcia Bertran W., Berberich M. J., Walker B. D., Riemer A. B., and Le Gall S. (2016) Analysis of major histocompatibility complex-bound HIV peptides identified from various cell types reveals common nested peptides and novel T cell responses. J. Virol. 90, 8605–8620 10.1128/JVI.00599-16 27440904 PMC5021429

[9] McMurtrey C., Trolle T., Sansom T., Remesh S. G., Kaever T., Bardet W., Jackson K., McLeod R., Sette A., Nielsen M., Zajonc D. M., Blader I. J., Peters B., and Hildebrand W. (2016) *Toxoplasma gondii* peptide ligands open the gate of the HLA class I binding groove. Elife 5, e12556 10.7554/eLife.12556 26824387 PMC4775218

[10] Bade-Döding C., Theodossis A., Gras S., Kjer-Nielsen L., Eiz-Vesper B., Seltsam A., Huyton T., Rossjohn J., McCluskey J., and Blasczyk R. (2011) The impact of human leukocyte antigen (HLA) micropolymorphism on ligand specificity within the HLA-B*41 allotypic family. Haematologica 96, 110–118 10.3324/haematol.2010.030924 20934997 PMC3012774

[11] Burrows S. R., Rossjohn J., and McCluskey J. (2006) Have we cut ourselves too short in mapping CTL epitopes? Trends Immunol. 27, 11–16 10.1016/j.it.2005.11.001 16297661

[12] Probst-Kepper M., Hecht H. J., Herrmann H., Janke V., Ocklenburg F., Klempnauer J., van den Eynde B. J., and Weiss S. (2004) Conformational restraints and flexibility of 14-meric peptides in complex with HLA-B*3501. J. Immunol. 173, 5610–5616 10.4049/jimmunol.173.9.5610 15494511

[13] Tynan F. E., Borg N. A., Miles J. J., Beddoe T., El-Hassen D., Silins S. L., van Zuylen W. J., Purcell A. W., Kjer-Nielsen L., McCluskey J., Burrows S. R., and Rossjohn J. (2005) High resolution structures of highly bulged viral epitopes bound to major histocompatibility complex class I. J. Biol. Chem. 280, 23900–23909 10.1074/jbc.M503060200 15849183

[14] Liu Y. C., Chen Z., Burrows S. R., Purcell A. W., McCluskey J., Rossjohn J., and Gras S. (2012) The energetic basis underpinning T-cell receptor recognition of a super-bulged peptide bound to a major histocompatibility complex class I molecule. J. Biol. Chem. 287, 12267–12276 10.1074/jbc.M112.344689 22343629 PMC3320977

[15] Guo H.-C., Jardetzky T. S., Garrett T. P., Lane W. S., Strominger J. L., and Wiley D. C. (1992) Different length peptides bind to HLA-Aw68 similarly at their ends but bulge out in the middle. Nature 360, 364–366 10.1038/360364a0 1448153

[16] Hassan C., Chabrol E., Jahn L., Kester M. G., de Ru A. H., Drijfhout J. W., Rossjohn J., Falkenburg J. H., Heemskerk M. H., Gras S., and van Veelen P. A. (2015) Naturally processed non-canonical HLA-A*02:01 presented peptides. J. Biol. Chem. 290, 2593–2603 10.1074/jbc.M114.607028 25505266 PMC4317018

[17] Rist M. J., Theodossis A., Croft N. P., Neller M. A., Welland A., Chen Z., Sullivan L. C., Burrows J. M., Miles J. J., Brennan R. M., Gras S., Khanna R., Brooks A. G., McCluskey J., and Purcell A. W., Rossjohn J., and Burrows S. R. (2013) HLA peptide length preferences control CD8+ T cell responses. J. Immunol. 191, 561–571 10.4049/jimmunol.1300292 23749632

[18] Achour A., Michaëlsson J., Harris R. A., Odeberg J., Grufman P., Sandberg J. K., Levitsky V., Kärre K., Sandalova T., and Schneider G. (2002) A structural basis for LCMV immune evasion: subversion of H-2Db and H-2Kb presentation of gp33 revealed by comparative crystal structure analysis. Immunity 17, 757–768 10.1016/S1074-7613(02)00478-8 12479822

[19] Li X., Lamothe P. A., Walker B. D., and Wang J.-H. (2017) Crystal structure of HLA-B*5801 with a TW10 HIV Gag epitope reveals a novel mode of peptide presentation. Cell. Mol. Immunol. 14, 631–634 10.1038/cmi.2017.24 28552904 PMC5520415

[20] Collins E. J., Garboczi D. N., and Wiley D. C. (1994) Three-dimensional structure of a peptide extending from one end of a class I MHC binding site. Nature 371, 626–629 10.1038/371626a0 7935798

[21] Tenzer S., Wee E., Burgevin A., Stewart-Jones G., Friis L., Lamberth K., Chang C. H., Harndahl M., Weimershaus M., Gerstoft J., Akkad N., Klenerman P., Fugger L., Jones E. Y., McMichael A. J., Buus S., Schild H., van Endert P., and Iversen A. K. (2009) Antigen processing influence HIV-specific cytotoxic T lymphocyte immunodominance. Nat. Immunol. 10, 636–646 10.1038/ni.1728 19412183

[22] Guillaume P., Picaud S., Baumgaertner P., Montandon N., Schmidt J., Speiser D. E., Coukos G., Bassani-Sternberg M., Filippakopoulos P., and Gfeller D. (2018) The C-terminal extension landscape of naturally presented HLA-I ligands. Proc. Natl. Acad. Sci. U.S.A. 115, 5083–5088 10.1073/pnas.1717277115 29712860 PMC5960288

[23] Komine-Aizawa S., Jiang J., Mizuno S., Hayakawa S., Matsuo K., Boyd L. F., Margulies D. H., and Honda M. (2019) MHC-restricted Ag85B-specific CD8+ T cells are enhanced by recombinant BCG prime and DNA boost immunization in mice. Eur. J. Immunol. 49, 1399–1414 10.1002/eji.201847988 31135967 PMC6722017

[24] Remesh S. G., Andreatta M., Ying G., Kaever T., Nielsen M., McMurtrey C., Hildebrand W., Peters B., and Zajonc D. M. (2017) Unconventional peptide presentation by major histocompatibility complex (MHC) class I HLA-A*02:01: breaking confinement. J. Biol. Chem. 292, 5262–5270 10.1074/jbc.M117.776542 28179428 PMC5392673

[25] Evnouchidou I., and van Endert P. (2019) Peptide trimming by endoplasmic reticulum aminopeptidase: role of MHC class I binding and ERAP dimerization. Hum. Immunol. 80, 290–295 10.1016/j.humimm.2019.01.003 30682405

[26] López de Castro J. A. (2018) How ERAP1 and ERAP2 shape the peptidome of disease-associated MHC I proteins. Front. Immunol. 9, 2463 10.3389/fimmu.2018.02463 30425713 PMC6219399

[27] Rock K. L., Reits E., and Neefjes J. (2016) Present yourself! By MHC class I and class II molecules. Trends Immunol. 37, 724–737 10.1016/j.it.2016.08.010 27614798 PMC5159193

[28] Howarth M., Williams A., Tolstrup A. B., and Elliott T. (2004) Tapasin enhances MHC class I peptide presentation according to peptide half-life. Proc. Natl. Acad. Sci. U.S.A. 101, 11737–11742 10.1073/pnas.0306294101 15286279 PMC511045

[29] Chen M., and Bouvier M. (2007) Analysis of interactions in a tapasin/class I complex provides a mechanism for peptide selection. EMBO J. 26, 1681–1690 10.1038/sj.emboj.7601624 17332746 PMC1829385

[30] Wearsch P. A., and Cresswell P. (2007) Selective loading of high-affinity peptides onto major histocompatibility complex class I molecules by the tapasin-ERp57 heterodimer. Nat. Immunol. 8, 873–881 10.1038/ni1485 17603487

[31] Rizvi S. M., and Raghavan M. (2010) Mechanism of function of tapasin, a critical major histocompatibility complex class I assembly factor. Traffic 11, 332–347 10.1111/j.1600-0854.2009.01025.x 20070606 PMC2983092

[32] Hammer G. E., Gonzalez F., Champsaur M., Cado D., and Shastri N. (2006) The aminopeptidase ERAAP shapes the peptide repertoire displayed by major histocompatibility complex class I molecules. Nat. Immunol. 7, 103–112 10.1038/ni1286 16299505

[33] Saveanu L., Carroll O., Lindo V., Del Val M., Lopez D., Lepelletier Y., Greer F., Schomburg L., Fruci D., Niedermann G., and van Endert P. M. (2005) Concerted peptide trimming by human ERAP1 and ERAP2 aminopeptidase complexes in the endoplasmic reticulum. Nat. Immunol. 6, 689–697 10.1038/ni1208 15908954

[34] Kanaseki T., Lind K. C., Escobar H., Nagarajan N., Reyes-Vargas E., Rudd B., Rockwood A. L., Van Kaer L., Sato N., Delgado J. C., and Shastri N. (2013) ERAPP and tapasin independently edit the amino and carboxyl termini of MHC class I peptides. J. Immunol. 191, 1547–1555 10.4049/jimmunol.1301043 23863903 PMC3735839

[35] Evnouchidou I., Weimershaus M., Saveanu L., and van Endert P. (2014) ERAP1-ERAP2 dimerization increases peptide-trimming efficiency. J. Immunol. 193, 901–908 10.4049/jimmunol.1302855 24928998

[36] Chen H., Li L., Weimershaus M., Evnouchidou I., van Endert P., and Bouvier M. (2016) ERAP1-ERAP2 dimers trim MHC I-bound precursor peptides; implications for understanding peptide editing. Sci. Rep. 6, 28902 10.1038/srep28902 27514473 PMC4981824

[37] Falk K., Rötzschke O., and Rammensee H.-G. (1990) Cellular peptide composition governed by major histocompatibility complex class I molecules. Nature 348, 248–251 10.1038/348248a0 2234092

[38] Kanaseki T., Blanchard N., Hammer G. E., Gonzalez F., and Shastri N. (2006) ERAAP synergizes with MHC class I molecules to make the final cut in the antigenic peptide precursors in the endoplasmic reticulum. Immunity 25, 795–806 10.1016/j.immuni.2006.09.012 17088086 PMC2746443

[39] Reeves E., Edwards C. J., Elliott T., and James E. (2013) Naturally occurring *ERAP1* haplotypes encode functionally distinct alleles with fine substrate specificity. J. Immunol. 191, 35–43 10.4049/jimmunol.1300598 23733883 PMC3785127

[40] Papakyriakou A., Reeves E., Beton M., Mikolajek H., Douglas L., Cooper G., Elliott T., Werner J. M., and James E. (2018) The partial dissociation of MHC class I-bound peptides exposes their N terminus to trimming by endoplasmic reticulum aminopeptidase 1. J. Biol. Chem. 293, 7538–7548 10.1074/jbc.RA117.000313 29599287 PMC5961055

[41] Phillips R. E., Rowland-Jones S., Nixon D. F., Gotch F. M., Edwards J. P., Ogunlesi A. O., Elvin J. G., Rothbard J. A., Bangham C. R., Rizza C. R., and McMichael A. J. (1991) Human immunodeficiency virus genetic variation that can escape cytotoxic T cell recognition. Nature 354, 453–459 10.1038/354453a0 1721107

[42] Reid S. W., McAdam S., Smith K. J., Klenerman P., O'Callaghan C. A., Harlos K., Jakobsen B. K., McMichael A. J., Bell J. I., Stuart D. I., and Jones E. Y. (1996) Antagonist HIV-1 gag peptides induce structural changes in HLA-B8. J. Exp. Med. 184, 2279–2286 10.1084/jem.184.6.2279 8976183 PMC2196387

[43] Bouvier M., Guo H.-C., Smith K. J., and Wiley D. C. (1998) Crystal structures of HLA-A*0201 complexed with antigenic peptides with either the amino-terminal group substituted by a methyl group. Proteins 33, 97–106 10.1002/(SICI)1097-0134(19981001)33:1<97::AID-PROT9>3.0.CO;2-I 9741848

[44] Garstka M. A., Fish A., Celie P. H., Joosten R. P., Janssen G. M., Berlin I., Hoppes R., Stadnik M., Janssen L., Ovaa H., van Veelen P. A., Perrakis A., and Neefjes J. (2015) The first step of peptide selection in antigen presentation by MHC class I molecules. Proc. Natl. Acad. Sci. U.S.A. 112, 1505–1510 10.1073/pnas.1416543112 25605945 PMC4321303

[45] Abualrous E. T., Saini S. K., Ramnarayan V. R., Ilca F. T., Zacharias M., and Springer S. (2015) The carboxy terminus of the ligand peptide determines the stability of the MHC class I molecule H-2Kb: a combined molecular dynamics and experimental study. PLoS One 10, e0135421 10.1371/journal.pone.0135421 26270965 PMC4535769

[46] Thomas C., and Tampé R. (2017) Structure of the TAPBPR-MHC I complex defines the mechanism of peptide loading and editing. Science 358, 1060–1064 10.1126/science.aao6001 29025996

[47] Jiang J., Natarajan K., Boyd L. F., Morozov G. I., Mage M. G., and Margulies D. H. (2017) Crystal structure of a TAPBPR-MHC I complex reveals the mechanism of peptide editing in antigen presentation. Science 358, 1064–1068 10.1126/science.aao5154 29025991 PMC6320693

[48] Dulberger C. L., McMurtrey C. P., Hölzemer A., Neu K. E., Liu V., Steinbach A. M., Garcia-Beltran W. F., Sulak M., Jabri B., Lynch V. J., Altfeld M., Hildebrand W. H., and Adams E. J. (2017) Human leukocyte antigen F presents peptides and regulates immunity through interactions with NK cell receptors. Immunity 46, 1018–1029.e7 10.1016/j.immuni.2017.06.002 28636952 PMC5523829

[49] Nguyen T. T., Chang S. C., Evnouchidou I., York I. A., Zikos C., Rock K. L., Goldberg A. L., Stratikos E., and Stern L. J. (2011) Structural basis for antigenic peptide precursor processing by the endoplasmic reticulum aminopeptidase ERAP1. Nat. Struct. Mol. Biol. 18, 604–613 10.1038/nsmb.2021 21478864 PMC3087843

[50] Zacharias M., and Springer S. (2004) Conformational flexibility of the MHC class I α1-α2 domain in peptide bound and free states: a molecular dynamics simulation study. Biophys. J. 87, 2203–2214 10.1529/biophysj.104.044743 15454423 PMC1304646

[51] Bailey A., van Hateren A., Elliott T., and Werner J. M. (2014) Two polymorphisms facilitate differences in plasticity between two chicken major histocompatibility class I proteins. PLoS One 9, e89657 10.1371/journal.pone.0089657 24586943 PMC3930747

[52] Thomas C., and Tampé R. (2017) Proofreading of peptide-MHC complexes through dynamic multivalent interactions. Front. Immunol. 8, 65 28228754 10.3389/fimmu.2017.00065 PMC5296336

[53] Blees A., Januliene D., Hofmann T., Koller N., Schmidt C., Trowitzsch S., Moeller A., and Tampé R. (2017) Structure of the human MHC I peptide-loading complex. Nature 551, 525–528 10.1038/nature24627 29107940

[54] Andrés A. M., Dennis M. Y., Kretzschmar W. W., Cannons J. L., Lee-Lin S. Q., and Hurle B., NISC Comparative Sequencing Program, Schwartzberg P. L., Williamson S. H., Bustamante C. D., Nielsen R., Clark A. G., and Green E. D. (2010) Balancing selection maintains a form of ERAP2 that undergoes nonsense-mediated decay and affects antigen presentation. PLoS Genet. 6, e1001157 10.1371/journal.pgen.1001157 20976248 PMC2954825

[55] Garboczi D. N., Hung D. T., and Wiley D. C. (1992) HLA-A2-peptide complexes: refolding and crystallization of molecules expressed in *Escherichia coli* and complexed with single antigenic peptides. Proc. Natl. Acad. Sci. U.S.A. 89, 3429–3433 10.1073/pnas.89.8.3429 1565634 PMC48881

[56] Otwinowski Z., and Minor W. (1997) Processing of X-ray diffraction data collected in oscillation mode. Methods Enzymol. 276, 307–326 10.1016/S0076-6879(97)76066-X 27754618

[57] Kabsch W. (2010) XDS. Acta Crystallogr. D Biol. Crystallogr. 66, 125–132 10.1107/S0907444909047337 20124692 PMC2815665

[58] McCoy A. J., Grosse-Kunstleve R. W., Adams P. D., Winn M. D., Storoni L. C., and Read R. J. (2007) Phaser crystallographic software. J. Appl. Crystallogr. 40, 658–674 10.1107/S0021889807021206 19461840 PMC2483472

[59] Adams P. D., Afonine P. V., Bunkóczi G., Chen V. B., Davis I. W., Echols N., Headd J. J., Hung L. W., Kapral G. J., Grosse-Kunstleve R. W., McCoy A. J., Moriarty N. W., Oeffner R., Read R. J., Richardson D. C., et al (2010) PHENIX: a comprehensive Python-based system for macromolecular structure solution. Acta Crystallogr. D Biol. Crystallogr. 66, 213–221 10.1107/S0907444909052925 20124702 PMC2815670

[60] Winn M. D., Murshudov G. N., and Papiz M. Z. (2003) Macromolecular TLS refinement in REFMAC at moderate resolutions. Methods Enzymol. 374, 300–321 10.1016/S0076-6879(03)74014-2 14696379

[61] Winn M. D., Ballard C. C., Cowtan K. D., Dodson E. J., Emsley P., Evans P. R., Keegan R. M., Krissinel E. B., Leslie A. G., McCoy A., McNicholas S. J., Murshudov G. N., Pannu N. S., Potterton E. A., Powell H. R., Read R. J., Vagin A., and Wilson K. S. (2011) Overview of the CCP4 suite and current developments. Acta Crystallogr. D Biol. Crystallogr. 67, 235–242 10.1107/S0907444910045749 21460441 PMC3069738

[62] Emsley P., and Cowtan K. (2004) Coot: model-building tools for molecular graphics. Acta Crystallogr. D Biol. Crystallogr. 60, 2126–2132 10.1107/S0907444904019158 15572765

