# Supplementary material for: ERAP1 enzyme-mediated trimming and structural analyses of MHC I–bound precursor peptides yield novel insights into antigen processing and presentation
Source: J Biol Chem. 2019 Oct 10;294(49):18534–44. doi: 10.1074/jbc.RA119.010102 (PMC6901306; doi:10.1074/jbc.RA119.010102)
Supplement: Supporting Information [file supp_294_49_18534__index.html]

ERAP1 enzyme-mediated trimming and structural analyses of MHC I­-bound precursor peptides yield novel insights into antigen processing and presentation — ERAP1 trimming and MHC I presentation of precursor peptides — ERAP1 enzyme-mediated trimming and structural analyses of MHC I–bound precursor peptides yield novel insights into antigen processing and presentation — EDITORS' PICK: ERAP1, MHC I, and precursor peptides — Supporting Information 

# ERAP1 enzyme-mediated trimming and structural analyses of MHC I–bound precursor peptides yield novel insights into antigen processing and presentation

## Supporting Information

- Figure S1 - This is a supporting Figure S1 (the only supporting figure in our manuscript)
