# Supplementary material for: ERAP1 enzyme-mediated trimming and structural analyses of MHC I–bound precursor peptides yield novel insights into antigen processing and presentation
Source: J Biol Chem. 2019 Oct 10;294(49):18534–44. doi: 10.1074/jbc.RA119.010102 (PMC6901306; doi:10.1074/jbc.RA119.010102)
Supplement: Supporting Information [file supp_RA119.010102_154176_1_supp_398205_py3cjj.pdf]

Supporting Information

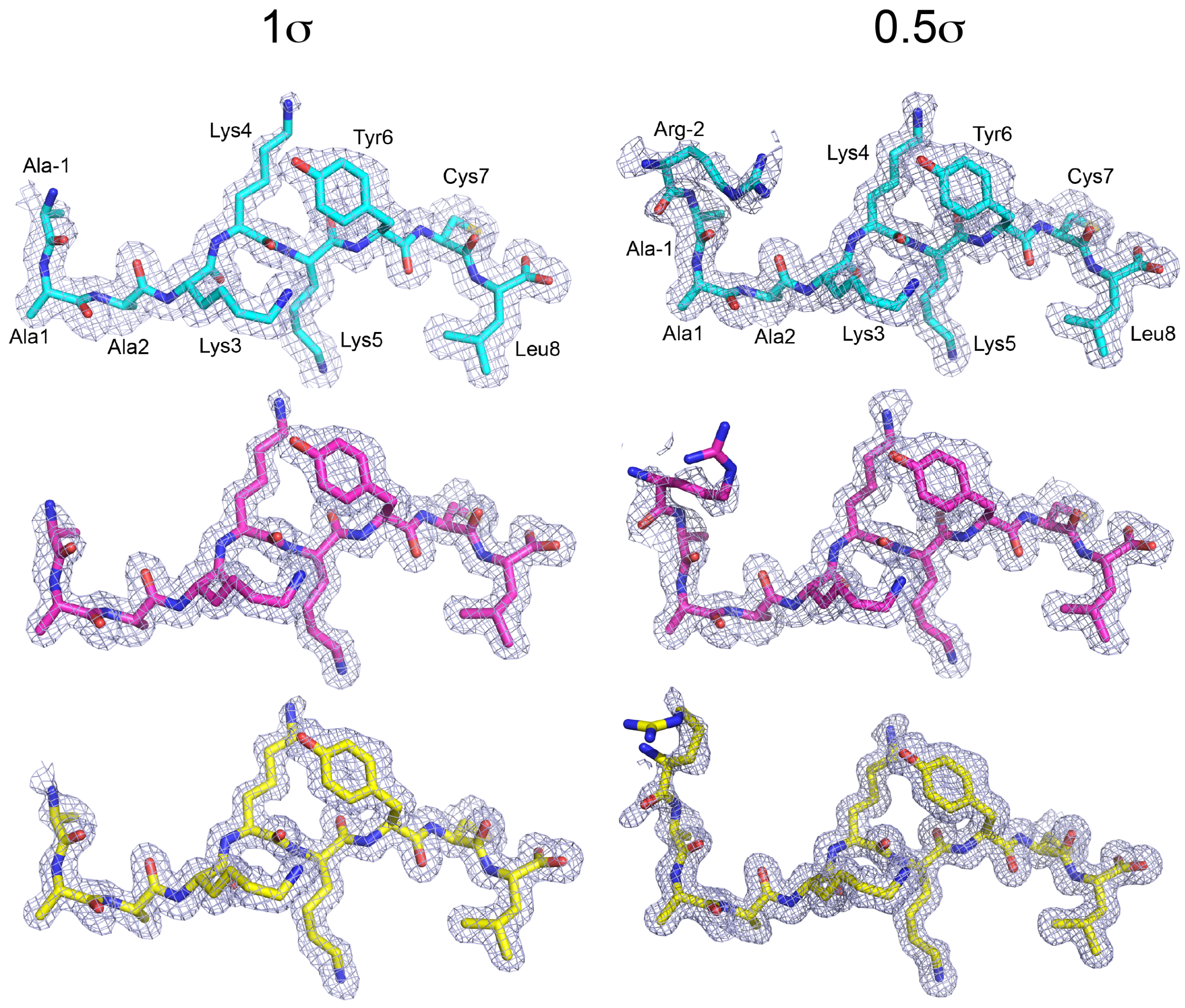

Figure S1. 2mFo-DFc electron density, contoured at 1σ (left panels) and 0.5σ (right panels), is shown as blue mesh around the 12 (cyan), 14 (magenta), and 20mer (yellow) (R(N-Me)A)$(RA)_{n-1}$AAKKKYCL. The density for P-2 Arg is partially visible at 0.5σ; we have omitted P-2 Arg and all residue extensions beyond P-2 from our final model. Peptide residues are labelled.
